# Does COVID‐19 pandemic impact cancer outcomes in metastatic setting? A comparative cohort study among metastatic patients treated at day care hospital

**DOI:** 10.1002/cam4.6378

**Published:** 2023-07-26

**Authors:** Florian Quilan, Justine Lequesne, François Cherifi, Etienne Bastien, Adeline Morel, Corinne Delcambre, Angélique Da Silva, Jean‐Michel Grellard, Alexandra Leconte, Audrey Faveyrial, Bénédicte Clarisse, Florence Joly

**Affiliations:** ^1^ Medical Oncology Department Centre François Baclesse Caen France; ^2^ Clinical Research Department Centre François Baclesse Caen France; ^3^ Anticipe (Interdisciplinary Research Unit for the Prevention and Treatment of Cancer), INSERM Unit 1086 Caen France

**Keywords:** COVID‐19, metastatic cancer, survival, treatment modification

## Abstract

**Introduction:**

COVID‐19 outbreak rapidly spread since early 2020 leading to the implementation of nationwide lockdowns. To cope with this sudden change, management guidelines were quickly published to adapt oncological care, with potential impact on cancer outcomes.

**Methods:**

We conducted a retrospective comparative cohort study to assess the impact of the COVID‐19 outbreak in 2020 on cancer outcomes in metastatic patients. Two cohorts of metastatic patients receiving intravenous (iv) therapy in a French oncological day care hospital were assessed: a 2020 cohort during the first French lockdown, and a 2018 historical cohort before the COVID‐19 pandemic. We performed a propensity score analysis to match patients from the two cohorts. After one‐year follow‐up, we compared progression‐free survival (PFS) and overall survival (OS) between cohorts. Adaptations of medical oncological treatments in 2020 were also analysed.

**Results:**

The 376 patients of the 2020 cohort were matched with 376 of the 2018 cohort. No SARS‐CoV‐2 infection was observed in the 2020 cohort. The adjusted PFS was significantly shorter in 2020 compared to 2018 (HR = 1.23; 95% CI: 1.03–1.46), as well as among patients without treatment adaptation compared to matched patients of the 2018 cohort (HR = 1.33; 95% CI: 1.10–1.61). We did not observe any significant difference of PFS among the group with treatment adaptations. OS was not significantly different.

**Conclusion:**

Metastatic cancer patients treated during the first lockdown had a higher risk of disease progression 1 year after COVID‐19 outbreak. However, oncological treatment adaptations or SARS‐CoV‐2 infections do not explain these results. A longer follow‐up is needed to observe the impact on OS.

## 1 INTRODUCTION

The new coronavirus disease 2019 (COVID‐19) first emerged in late 2019 in Wuhan, China, and rapidly grew into a global pandemic. As of late 2022, there were over 634 million confirmed cases of COVID‐19 and 6.6 million deaths reported.^1^ In France, the first severe acute respiratory syndrome coronavirus 2 (SARS‐CoV‐2) infection was diagnosed in January 2020.^2^ The pandemic rapidly spread afterward, leading to the implementation of a first nationwide lockdown from 17 March to 11 May, 2020.

Several studies have reported a higher risk of SARS‐CoV‐2 infection together with more related complications for cancer patients, notably in case of progressive disease.^3^, ^4^, ^5^, ^6^, ^7^, ^8^, ^9^ In response to both the increased vulnerability to SARS‐CoV‐2 and the lockdown measures, guidelines were rapidly issued to adapt oncological care during the COVID‐19 pandemic.^10^, ^11^, ^12^ In particular, hospital admissions were avoided and management at home was encouraged. In this context, the use of telemedicine instead of on‐site monitoring visits was encouraged, as well as replacement of intravenous drugs with oral drugs, adjustment of chemotherapy regimens or shortening of radiotherapy treatments. Moreover, patients with slowly evolving metastatic cancers could have temporary breaks in their treatment. Besides, when healthcare facilities were challenged, priority was given to patients either with curative‐intent therapeutic strategies or with the longest life expectancy. Furthermore, cancer supportive care was adapted in order to avoid hospitalisations due to oncological treatment complications (e.g. extended use of granulocyte‐colony stimulating factor in primary prophylaxis).^13^, ^14^

Due to these pandemic‐related adaptations, cancer outcomes may have been affected, but remain poorly documented to date.^15^ In particular, there are no data dedicated to prognostic of metastatic cancer patients managed during the COVID‐19 pandemic. Yet, it may be assumed that these patients were particularly vulnerable to the effects of COVID‐19 on the healthcare pathway and, as a consequence, more likely to progress in the short to medium term.

We have previously conducted a prospective study (NCT04366154) in adult patients with solid/hematologic malignancy with ongoing medical treatment during the first COVID‐19 lockdown in the outpatient clinics of two French comprehensive cancer centres. It highlighted that medical oncology practice was modified for 27% of the 734 patients enrolled, especially 29% for the 435 patients in metastastic setting.^16^ Clinical outcomes of these medical adjustments were not investigated. In this context, we aimed to assess the impact of the COVID‐19 outbreak on cancer outcomes in metastatic patients undergoing an anticancer treatment per intravenous (iv) route in the day care hospital, as compared with a historical cohort of metastatic patients treated before the COVID‐19 pandemic.

## 2 METHODS

### 2.1 Study design and patients

This study is a retrospective comparative cohort study of adults with metastatic solid tumour who received an iv anti‐cancer treatment (either chemotherapy, immunotherapy and/or targeted therapy) at the day care hospital of the comprehensive cancer Centre François Baclesse, Caen, France. Patients receiving exclusive oral therapy or already participating in a clinical trial were not eligible. The study compared a cohort of patients treated during the first nationwide COVID‐19 lockdown from March to June 2020 (the 2020 cohort) to a historical cohort of patients treated from March to June 2018 (the 2018 cohort) in the outpatient department.

The 2020 cohort consisted of patients from the COVIPACT study who met the eligibility criteria. The COVIPACT study was a one‐year longitudinal prospective study initiated at the beginning of the first French nationwide lockdown: it aimed to assess adjustments in oncological practice, as well as psychological consequences.^16^ It enrolled 734 participants who were at least 18 years old, were treated for solid or hematologic cancer in two regional cancer centres during the first COVID‐19 lockdown (Clinical trial NCT04366154), of whom 435 patients were in metastatic setting.

The 2018 historical cohort was constituted retrospectively from medical records of the day care hospital. It included all eligible metastatic patients receiving an anti‐cancer treatment per iv route between March and June 2018.

### 2.2 Data collection

This retrospective study was approved by the institutional review board. It was conducted in compliance with the French Research Standard MR‐004 ‘Research not involving Human participants’ (compliance commitment to MR‐004 for the Centre François Baclesse number 2214228 v.0, dated from 07/03/2019). It is registered with the French Health Data Hub under the reference F20210415165210. All patients received information and none of them expressed opposition to the use of their data.

Collected data included sociodemographic parameters and clinical information, such as patient characteristics, cancer diagnosis and treatments, including ongoing treatment at the time of inclusion (between March and June for each cohort), clinical cancer outcomes and survivals. For the 2020 cohort, data were extracted from the database of the COVIPACT study for patients. They also included information on adjustments in oncological treatments, based on the physician decision after a multidisciplinary committee discussion considering clinical characteristics of the patients, ongoing treatment and status of the disease. Survival outcomes (disease progression and/or death) were also collected within 1 year after inclusion for the purpose of our study. For the 2018 cohort, data were collected from the registry database and clinical records of patients.

### 2.3 Study purposes and statistical analysis

The primary endpoint was to compare the progression‐free survival (PFS) of patients between the 2020 cohort and the 2018 cohort, with a one‐year follow‐up. Secondary objectives were to assess and compare overall survival between the two cohorts, and to evaluate the PFS outcomes of patients with and without cancer treatment adaptations in the 2020 cohort, as compared to matched patients in the 2018 cohort.

Data were described by using number and proportion for categorical variables, mean and standard deviation for continuous variables. Comparisons of patient's characteristics between cohorts were assessed by the use of Pearson's chi‐squared test for categorical data, and by independent sample *t*‐tests for continuous data.

PFS was defined as the time elapsed from the patient's start date of the ongoing treatment at inclusion to disease progression, or death whatever the cause. OS was defined as the time elapsed from the patient's start date of the ongoing treatment at inclusion to death. Patients who remained without an event by the end of the study period (30 June 2019 for the 2018 cohort and 30 June 2020 for the 2020 cohort) were censored. PFS and OS were estimated by the Kaplan–Meier method. Univariate and multivariable Cox models were performed to measure the difference of survival outcomes between cohorts with adjustment on patient's characteristics and treatment (age, sex, body mass index (BMI), performance status, primary tumour location, number of treatment lines, and treatment protocol (distinguishing four categories: chemotherapy, chemotherapy + targeted therapy, immunotherapy, targeted therapy); time from diagnosis being closely related to number of treatment lines was not included in the multivariable model).

To reduce the effects of confounding variables due to the non‐randomised data in the estimation of COVID‐19 pandemic on survival outcomes, we conducted a propensity score matching. The propensity score, here defined as the probability of cohort assignment, was estimated by using a multivariable logistic regression model, containing age, sex, primitive tumour location, European Cooperative Oncology Group Performance Status (ECOG PS) and treatment protocol. Patients of the 2020 cohort were matched with patients of the 2018 cohort in a 1:1 ratio, without replacement, by using the nearest neighbour method. Additionally, separated analyses were then conducted among patients for which treatment was adjusted in 2020 due to the pandemic, and patients for which treatment was administered as usual, each of these two sub‐samples being matched with samples of the 2018 cohort.

All statistical analyses were carried out using R statistical software (4.1.2). A two‐side *p*‐value less than 0.05 was considered statistically significant.

## 3 RESULTS

### 3.1 Population description

#### 3.1.1 Screened population

**FIGURE 1:**
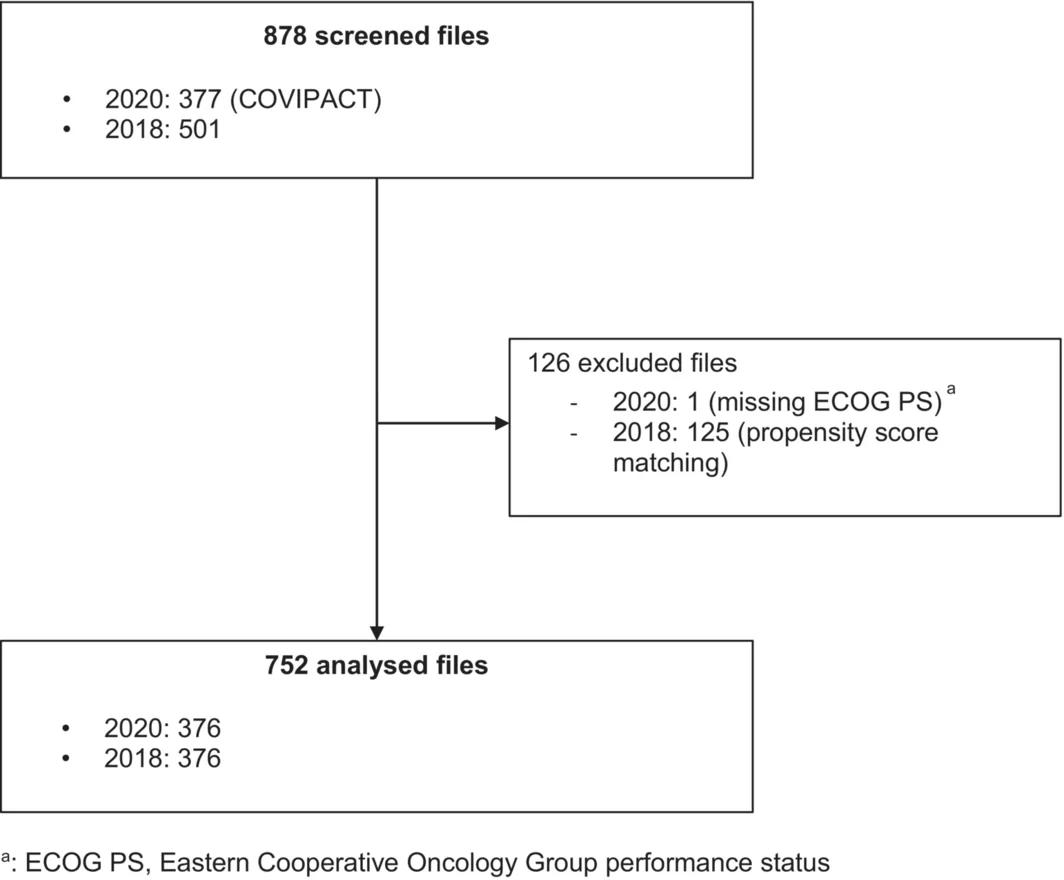
Study flow chart.

Among the 435 patients in metastatic setting enrolled in the COVIPACT study, 377 patients undergoing iv treatment at day care hospital of the Centre François Baclesse between March and June 2020 met the eligibility criteria. From medical records of our centre, 501 metastatic patients treated with iv treatment at day care hospital at the same period in 2018 were identified (Figure 1). The sociodemographic and clinical characteristics of these patients are presented in Table S1.

#### 3.1.2 Analysed population

Propensity score analysis including age, sex, primitive tumour location, ECOG PS and treatment protocol allowed to match 376 patients of the 2020 cohort with 376 patients of the 2018 cohort, the remaining patient of the 2020 cohort being not matched because of missing ECOG PS (Figure 1). The sociodemographic and clinical characteristics were globally balanced between the two cohorts (Table 1). Women accounted for 61.8% of patients, the median age was 65.4 years and the three main cancer locations were breast (31.1%), digestive (25.5%) and lung (20.9%). Most of the patients had an ECOG PS of 0 or 1 (87.4%) and were in first or second line of treatment (76.6%). Chemotherapy with or without targeted therapy was the most prescribed treatment (70.2%), followed by targeted therapy alone (17.7%) and immunotherapy (12.1%).

**TABLE 1:** Characteristics of the analysed population (*N* = 752).

	2018 cohort *N* = 376		2020 cohort *N* = 376		*p* value
Gender					0.37
Women	239	(63.6%)	226	(60.1%)	
Men	137	(36.4%)	150	(39.9%)	
Age at inclusion (years)					
Median (Q1–Q3)	65.4	(57.6–71.6)	65.4	(58.5–71.8)	0.6
>70	122	(32.4%)	126	(33.5%)	0.82
Diagnostic delay (months)					
Median (Q1–Q3)	13.4	(4.5–30.2)	14.9	(5.4–33.4)	0.21
>24	126	(33.5%)	127	(33.8%)	1
Location					0.064
Breast	126	(33.5%)	108	(28.7%)	
Digestive	100	(26.6%)	92	(24.5%)	
Gynaecological	38	(10.1%)	38	(10.1%)	
Lung	75	(19.9%)	82	(21.8%)	
Urogenital	25	(6.6%)	25	(6.6%)	
Head and neck	12	(3.2%)	31	(8.2%)	
Performance status					0.83
ECOG PS 0–1	330	(87.8%)	327	(87%)	
ECOG PS 2–3	46	(12.2%)	49	(13%)	
BMI (kg/m^2^)					0.36
Median (Q1–Q3)	24.5	(21–27.7)	24.7	(21.6–27.9)	
Ongoing treatment at inclusion					0.71
Line 1	185	(49.2%)	193	(51.3%)	
Line 2	98	(26.1%)	100	(26.6%)	
Line 3	46	(12.2%)	46	(12.2%)	
Line ≥4	47	(12.5%)	37	(9.8%)	
Ongoing treatment protocol					0.38
Chemotherapy	195	(51.9%)	180	(47.9%)	
Chemotherapy + targeted therapy	77	(20.5%)	76	(20.2%)	
Immunotherapy	38	(10.1%)	53	(14.1%)	
Targeted therapy	66	(17.6%)	67	(17.8%)	

Abbreviations: BMI, body mass index; ECOG PS, Eastern Cooperative Oncology Group Performance Status.

None of the enrolled patients experienced a SARS‐CoV‐2 infection and no patient died from COVID‐19 during the first French lockdown.

#### 3.1.3 Treatment adaptations

In the 2020 cohort, 104 patients (27.6%) had an adaptation of their medical oncology program, including adjournments or treatment interruptions (37.5% and 23.1% respectively), modified treatment plans (33.7%), modified treatment type (4.8%), modified treatment administration method (2.9%) or adapted monitoring (9.6%).

The characteristics of the patients with treatment adaptations are presented in Table 2. In comparison to the whole 2020 cohort, the subgroup with treatment adaptations was particularly different. Breast cancer was still the most represented but with a greater proportion (37.5% vs. 28.7%). Lung and head and neck cancers were also more represented (31.7% and 12.5%, respectively vs. 21.8% and 8.2%), whereas digestive and gynaecological cancers were less concerned (6.7% both vs. 24.5% and 10.1%, respectively). Patients were still mostly in first line of treatment but with a higher predominance on the other lines (66.3% vs. 51.3%). While 68.1% of patients included in the 2020 cohort received chemotherapy with or without targeted therapy, only 36.5% of those with treatment adaptations were treated with these types of treatment. Targeted therapy alone (e.g. Trastuzumab) and immunotherapy alone (e.g. Pembrolizumab) were the main treatment protocols (40.4% and 23.1%, respectively vs. 17.8% and 14.1%).

**TABLE 2:** Characteristics of patients of the 2020 cohort according to pandemic‐induced treatment adaptations (*N* = 376).

	Presence of adaptations (*N* = 103)		Absence of adaptations (*N* = 273)		*p* value
Gender					0.12
Women	69	67%	157	57.5%	
Men	34	33%	116	42.5%	
Age at inclusion (years)					
Median (Q1–Q3)	64.3	(58.9–70.4)	65.8	(58.1–72.1)	0.4
>70	26	25.2%	100	36.6%	**0.05**
Diagnostic delay (months)					
Median (Q1–Q3)	19.7	(10.1–44.0)	12.9	(4.5–30.1)	**<0.001**
>24	46	44.7%	81	29.7%	**0.009**
Location					**<0.001**
Breast	39	37.9%	69	25.3%	
Digestive	6	5.8%	86	31.5%	
Gynaecological	7	6.8%	31	11.4%	
Lung	33	32%	49	17.9%	
Urogenital	5	4.9%	20	7.3%	
Head and neck	13	12.6%	18	6.6%	
Performance status					0.98
ECOG PS 0–1	89	86.4%	238	87.2%	
ECOG PS 2–3	14	13.6%	35	12.8%	
BMI (kg/m^2^)					0.34
Median (Q1–Q3)	24	(21.5–27.9)	25	(21.9–27.9)	
Ongoing treatment line at inclusion					**<0.001**
Line 1	69	67%	124	45.4%	
Line 2	22	21.4%	78	28.6%	
Line 3	9	8.7%	37	13.6%	
Line ≥4	3	2.9%	34	12.5%	
Ongoing treatment protocol					**<0.001**
Chemotherapy	25	24.3%	155	56.8%	
Chemotherapy and targeted therapy	12	11.7%	64	23.4%	
Immunotherapy	24	23.3%	29	10.6%	
Targeted therapy	42	40.8%	25	9.2%	

*Note*: In bold significant results. Other treatment includes any combinations of immunotherapy or endocrine therapy with other therapy (all administered to <2%).

Abbreviation: BMI, body mass index.

The characteristics of the subgroup without treatment adaptation were more similar to the whole 2020 cohort, especially concerning the proportion of digestive cancers and chemotherapy (Table 2).

### 3.2 Progression free survival

The one‐year PFS rate was 28.7% (95% CI: 24.5–33.7) in the 2020 cohort and 30.3% (95% CI: 26.0–35.3) in the 2018 cohort.

In the multivariable model, after adjustment on sex, ECOG PS, number of treatment lines, cancer location and treatment protocol as well as age and BMI, PFS was significantly decreased in the 2020 cohort (HR = 1.23; 95% CI: 1.03–1.46; *p* = 0.019; Table 3).

**TABLE 3:** Univariable and multivariable analysis for PFS in the analysed population.

	Univariable analysis			Multivariable analysis		
	HR	95% CI	*p* value	HR	95% CI	*p* value
Gender Reference: Female	1.63	[1.37–1.94]	**<0.001**	1.27	[1.00–1.60]	**0.047**
Performance status Reference: ECOG PS 0–1	1.44	[1.13–1.84]	**0.003**	1.35	[1.04–1.73]	**0.026**
BMI (kg/m^2^)			0.31			0.53
0–20	1	–		1	–	
20–25	0.87	[0.68–1.11]		0.89	[0.69–1.16]	
25 +	0.83	[0.65–1.05]		0.87	[0.67–1.11]	
Age at inclusion (years) Reference: <70	1.14	[0.95–1.36]	0.16	1.05	[0.88–1.26]	0.59
Time from diagnosis (months) Reference: <24	0.77	[0.64–0.93]	**0.007**			
Number of treatment lines	1.28	[1.21–1.36]	**<0.001**	1.24	[1.16–1.33]	**<0.001**
Primary tumour localisation			**<0.001**			**0.004**
Breast	1	‐		1	‐	
Digestive	1.88	[1.49–2.36]		1.55	[1.12–2.15]	
Gynaecological	1.3	[0.94–1.80]		1.24	[0.89–1.75]	
Lung	1.43	[1.11–1.84]		1.82	[1.30–2.55]	
Urogenital	1.61	[1.12–2.32]		1.43	[0.92–2.23]	
Head and neck	2.33	[1.61–3.37]		2.44	[1.53–3.89]	
Ongoing treatment protocol			**<0.001**			**<0.001**
Chemotherapy	1	‐		1	‐	
Chemo + targeted therapy	0.78	[0.64–0.97]		0.77	[0.61–0.98]	
Immunotherapy	0.35	[0.26–0.49]		0.27	[0.19–0.39]	
Targeted therapy	0.2	[0.15–0.28]		0.31	[0.22–0.45]	
Time period (2018 vs. 2020) Reference: 2018	1.12	[0.95–1.33]	0.18	1.23	[1.03–1.46]	**0.019**

*Note*: In bold significant results.

Abbreviations: BMI, body mass index; PFS, progression‐free survival.

In the univariable analysis, male gender (*p* < 0.001), ECOG PS >1 (*p* = 0.003), number of treatment lines (*p* < 0.001), primary tumour location (*p* < 0.001) and treatment protocol (*p* < 0.001) were associated with a significantly shorter PFS. Time from diagnosis > 24 months (*p* = 0.007) was associated with a significantly longer PFS. According to the period, there was no significant difference between the two cohorts (HR = 1.12; 95% CI: 0.95–1.33; *p* = 0.18; Table 3; Figure S1).

#### 3.2.1 Treatment adaptations

One hundred three patients with pandemic‐induced treatment adaptations of the 2020 cohort were matched with 103 patients of the 2018 cohort. After adjustment on sex, ECOG PS, BMI, age, number of treatment lines, cancer location and treatment protocol, there was no significant difference in PFS according to time period (HR = 0.71; 95% CI: 0.48–1.07; *p* = 0.10; Table 4).

**TABLE 4:** PFS (multivariable model) for matched samples of patients of the 2020 cohort with or without treatment adaptations with patients of the 2018 cohort.

	Matched samples ‐ treatment adaptations in 2020 (*N* = 206)^a^			Matched samples – no treatment adaptations in 2020 (*N* = 546)^b^		
	HR	95% CI	*p*	HR	95% CI	*p*
Gender Reference: Female	1.30	[0.77–2.18]	0.33	1.31	[1.0–1.72]	**0.042**
Performance status Reference: ECOG PS 0–1	1.66	[0.98–2.83]	0.072	1.22	[0.91–1.64]	0.19
BMI			0.32			0.34
0–20	1	‐		1	‐	
20–25	0.81	[0.48–1.36]		0.89	[0.67–1.19]	
25 +	0.66	[0.39–1.13]		0.81	[0.61–1.08]	
Age at inclusion (years) Reference: <70	0.92	[0.59–1.44]	0.72	1.00	[0.81–1.23]	0.98
Number of treatment lines	1.12	[0.92–1.36]	0.27	1.29	[1.20–1.39]	**<0.001**
Primary tumour localisation			0.037			**0.008**
Breast	1	‐		1	‐	
Digestive	1.85	[0.64–5.40]		1.46	[1.02–2.10]	
Gynaecological	2.85	[1.29–6.29]		0.97	[0.67–1.40]	
Lung	3.19	[1.46–6.96]		1.84	[1.24–2.73]	
Urogenital	2.19	[0.60–8.00]		1.89	[1.15–3.11]	
Head and neck	3.26	[1.26–8.46]		2.57	[1.49–4.42]	
Ongoing treatment protocol			**<0.001**			**<0.001**
Chemotherapy	1	‐		1	‐	
Chemo + targeted therapy	0.96	[0.52–1.77]		0.81	[0.62–1.06]	
Immunotherapy	0.24	[0.12–0.45]		0.32	[0.21–0.49]	
Targeted therapy	0.42	[0.21–0.81]		0.38	[0.24–0.61]	
Time period (2018 vs. 2020) Reference: 2018	0.71	[0.48–1.07]	0.10	1.33	[1.10–1.61]	**0.004**

*Note*: In bold significant results.

Abbreviation: BMI, body mass index; PFS, progression‐free survival.

^a^ 103 patients in the 2020 cohort with treatment adaptations matched to another group of 103 patients from the 2018 cohort.

^b^ 273 patients in the 2020 cohort without treatment adaptations matched to another group of 273 patients from the 2018 cohort.

For patients without treatment adaptation, using the same matching and adjustment, we found a significant difference in PFS according to time period, with a higher risk of progression in 2020 (HR = 1.33; 95% CI: 1.10–1.61; *p* = 0.004; Table 4).

### 3.3 Overall survival

The one‐year OS rate was 66% (95% CI: 61.3–70.9) for the 2020 cohort and 64.9% (95% CI: 60.2–69.9) for the 2018 cohort.

There was no difference according to the time period in the univariate analysis (HR = 0.99; 95% CI: 0.77–1.26; *p* = 0.92; Table S2; Figure S2), as well as in the multivariable model (HR = 0.91; 95% CI: 0.71–1.16; *p* = 0.44; Table 2).

## 4 DISCUSSION

In this retrospective comparative cohort study, we investigated for the first time the impact of COVID‐19 outbreak on cancer outcomes among metastatic patients undergoing iv treatment at a day care hospital. With one‐year follow‐up, the adjusted PFS was significantly shorter in the 2020 cohort compared to the 2018 cohort. In the subgroup of patients without cancer treatment adaptation due to the pandemic, PFS was shorter as well. None of the patients included in 2020 was infected by SARS‐CoV‐2 and no difference of PFS was observed among the subgroup with treatment adaptations as compared to a matched sample of the 2018 cohort. OS was not significantly different between the two cohorts.

Many studies have focused on the link between SARS‐CoV‐2 infections and cancer‐related deaths in 2020.^17^, ^18^, ^19^ For example, in a French study, there were poorer OS rates in 2020 for patients diagnosed with colorectal cancer and infected with SARS‐CoV‐2, compared to 2018 and 2019.^20^ In contrast to these studies, the COVID‐19 pandemic was not involved in the survival outcomes of our patients. This may be partly explained by the fact that no patients in the 2020 cohort experienced SARS‐CoV‐2 infection during the first French lockdown. The absence of SARS‐CoV‐2 infection may result from the implementation of adapted healthcare pathway for these patients at higher risk of contamination, while limiting hospital admissions and favouring management at home. Another before‐after study conducted in Portugal enrolled patients diagnosed with cancer during the first lockdown period in 2020 with different cancer locations and compared them to patients diagnosed in the same period of 2019. Among the 763 patients diagnosed in 2020, only nine were infected by SARS‐CoV‐2. After a follow‐up of 4 months, there was an increase in overall short‐term mortality of diagnosed cancer patients after COVID‐19 outbreak, especially for patients with stage III cancer and those undergoing surgical treatment or radiotherapy.^21^ Increase of short‐term mortality was partly explained by adaptations of healthcare system during the COVID‐19 pandemic. Indeed, diagnosis delays were observed in the first half of 2020 as well as presentations with more clinically advanced conditions.^22^ Furthermore, some modelling studies have predicted a long‐term effect on survival outcomes, up to 5 years.^23^, ^24^

In our study, we included metastatic cancer patients undergoing iv treatment, which represents a different oncology population. With a longer follow‐up of 1 year, OS was not significantly different after the COVID‐19 outbreak. Nevertheless, there was a higher risk of disease progression in the 2020 cohort as well as in the subgroup without treatment adaptation, possibly because of similar characteristics as compared to the 2018 cohort. At first, similarly to the Portuguese study, changes in access to care may be involved, especially due to delays in routine follow‐up reported in 2020.^25^ Follow‐up of metastatic disease may have been impaired by the pandemic, with, for example, delays in follow‐up imaging or appointments. Hospital avoidance may also have caused patients to wait to seek medical attention in case of symptoms. Moreover, psychosocial effects of COVID‐19 outbreak on cancer patients may also be considered. Indeed, anxiety and depression symptoms during the COVID‐19 pandemic have been reported in several studies from different countries.^16^, ^26^, ^27^ The potential role of psychosocial factors on cancer outcomes has been studied. In a meta‐analyse, chronic daily life event stress, severe life events, depression and social isolation favoured cancer growth and metastasis, concluding that psychosocial factors may represent risk factors for cancer incidence and evolution.^28^

Reassuringly, we did not observe significant change in PFS for patients in metastatic setting with adapted treatment in 2020. This probably reflects the relevance of guidelines and appropriate patient identification for implementation of adapted cancer management. According to guidelines, if the healthcare system was overwhelmed, a triaging could be done to prioritise oncological emergencies and curative treatments over palliative treatment for advanced pathologies.^11^, ^29^ In other cases, the adaptations in oncological treatment program were weighted by the need to continue therapies aiming at curing and treating cancer, as mortality from untreated active malignancy remains extremely high and by the modest risk of severe COVID‐19 due to systemic anticancer therapy.^30^, ^31^ In an American cohort study that focused on metastatic solid cancers, there were no delays in systemic treatment initiation or preference against use of myelosuppressive therapies.^32^ In our study, capacities of the day care hospital, although modified, were not overwhelmed and adaptations of treatment plans were made for a selected group of metastatic patients of good prognosis (mainly monotherapy with immunotherapy/targeted therapy for patients with a good performance status) while patients with a poorer prognosis treated with chemotherapy or combined treatments continued their program. Likewise, in other studies, short‐term cancer outcomes have not been worsened by modifications of treatment plans.^20^, ^33^ Similarly, recent observations by Tran et al. from a large retrospective cohort addressing the impact of the COVID‐19‐related health care disruptions on pathologic cancer staging: no statistically significant differences in pathologic stage or features between cancers staged in the first year of the pandemic as compared with cancers staged in the 2 years before the pandemic were found.^15^

These various findings addressing different facets of cancer care pathway thus provide reassuring insight into cancer care patterns during the pandemic, from diagnosis to management of metastatic cancer patients. They support the insightfulness of the recommendations by learned cancer societies for adaptations of cancer care to cope with such a pandemic. They also demonstrate that despite disturbances in delivery of health care services, the prioritisation of cancer procedures during times of reduced capacity allowed to maintain a similar quality of cancer management.

We constituted a large cohort of 752 patients with multiple cancer sites, which is, to our knowledge, the first to explore the impact of COVID‐19 outbreak on cancer outcomes of metastatic patients undergoing an iv treatment at day care hospital. In contrast to many other studies evaluating the relationship between cancer and SARS‐CoV‐2 infections, there was no patient infected in our study, which allowed us to exclude any involvement on cancer outcomes. Although it was not feasible to realise a ‘gold standard’ control study without COVID‐19 involvement, we formed a historical cohort consisting of patients treated in 2018 over the same period as the 2020 cohort. A propensity score matching was made to circumvent the lack of randomisation between the two groups and to enhance comparability between them.

However, this study has some limitations. First, our data came from a single French cancer centre so that it could not reflect great variations of the SARS‐Cov‐2 infection rate and healthcare overwhelming by regions and countries. In addition, we did not collect information on socioeconomic status of patients, or other relevant characteristics, including qualitative data, that may have provided some additional relevant context and may have been helpful to nuance the interpretation of the findings. Moreover, we collected whether patients had progressed or died but we did not collect disease‐related or treatment‐related complications that may have impacted PFS. In addition, the follow‐up of our study was short and a longer follow‐up seems necessary to assess further information on long‐term survival outcomes. The natural evolution of the pandemic and the implementation of vaccination campaigns since 2021 should also be considered.

## 5 CONCLUSION

In this comparative retrospective cohort study, the adjusted PFS at one‐year follow‐up was significantly shorter in the 2020 cohort compared to our historical 2018 cohort. PFS was similarly shorter in the subgroup without treatment adaptation, arguing for a potential effect of the pandemic on metastatic disease progression of patients receiving iv treatment at day care hospital. The changes in access to care and psychosocial issues may be involved. In the subgroup with treatment pandemic‐related adaptations in 2020, PFS was no more significantly different. The impact of treatment adaptations was reassuringly limited by following relevant guidelines to select patients with a better prognosis. OS was also not significantly different. Our findings could serve as valuable feedback in the event of future pandemic. Furthermore, it may be also relevant to involve patient advocacy groups in discussions to better integrate the patient perspective in the care prioritisation and to facilitate patient's adhesion to the therapeutic options and care management. Nevertheless, a longer follow‐up is necessary to better understand the impact on long‐term survival outcomes.

## AUTHOR CONTRIBUTIONS

**Florian Quilan:** Conceptualization (equal); investigation (equal); methodology (equal); validation (equal); writing – original draft (lead); writing – review and editing (equal). **Justine Lequesne:** Conceptualization (equal); formal analysis (equal); methodology (equal); writing – original draft (equal); writing – review and editing (equal). **François Cherifi:** Writing – review and editing (equal). **Etienne Bastien:** Writing – review and editing (equal). **Adeline Morel:** Writing – review and editing (equal). **Corinne Delcambre:** Writing – review and editing (equal). **Angélique Da Silva:** Writing – review and editing (equal). **Jean‐Michel Grellard:** Project administration (lead); writing – review and editing (equal). **Alexandra Leconte:** Project administration (equal); writing – review and editing (equal). **Audrey Faveyrial:** Writing – review and editing (equal). **Bénédicte Clarisse:** Funding acquisition (lead); methodology (equal); project administration (equal); supervision (equal); validation (equal); writing – original draft (equal); writing – review and editing (equal). **Florence Joly:** Conceptualization (lead); methodology (equal); supervision (lead); validation (equal); writing – original draft (equal); writing – review and editing (equal).

## FUNDING INFORMATION

The COVIPACT trial was supported by a research grant from Fondation ARC (COVID202001320) and financial support from the GEFLUC Normandie (Les Entreprises Contre le Cancer/Campaigns Against Cancer, Rouen‐Normandie). No additional funding was obtained for this retrospective comparative cohort study. The funders had no role in the design of the study, the collection, analysis, and interpretation of the data, the writing of the manuscript, and the decision to submit the manuscript for publication.

## CONFLICT OF INTEREST STATEMENT

Authors declare no conflict of interest.

## ETHICS STATEMENT

All data used in the study have been pseudonymized. This study was approved by the institutional review board. It was conducted in accordance with the French legislation and with compliance with the French Research Standard MR‐004 ‘Research not involving Human participants’ (compliance commitment to MR‐004 for the Centre François Baclesse, CNIL number 2214228 v.0, dated from 07/03/2019). It is registered with the French Health Data Hub under the reference F20210415165210 (https://www.health‐data‐hub.fr/projets/covipact‐obs‐etude‐de‐cas‐temoins‐evaluant‐limpact‐de‐la‐situation‐de‐pandemie‐de‐covid19). All patients received information and none of them expressed opposition to the use of their data.

## Supporting information


Figure S1.

Figure S2.

Table S1.

Table S2.

## Data Availability

The data that support the findings of this study are available upon reasonable request to the corresponding author.
