# Supplementary material for: Does COVID‐19 pandemic impact cancer outcomes in metastatic setting? A comparative cohort study among metastatic patients treated at day care hospital
Source: Cancer Med. 2023 Jul 26;12(17):17603–12. doi: 10.1002/cam4.6378 (PMC10523941; doi:10.1002/cam4.6378)

Supplementary figure 2. Overall survival of the 2020 treated cohort compared to the 2018 treated cohort (univariable analysis)

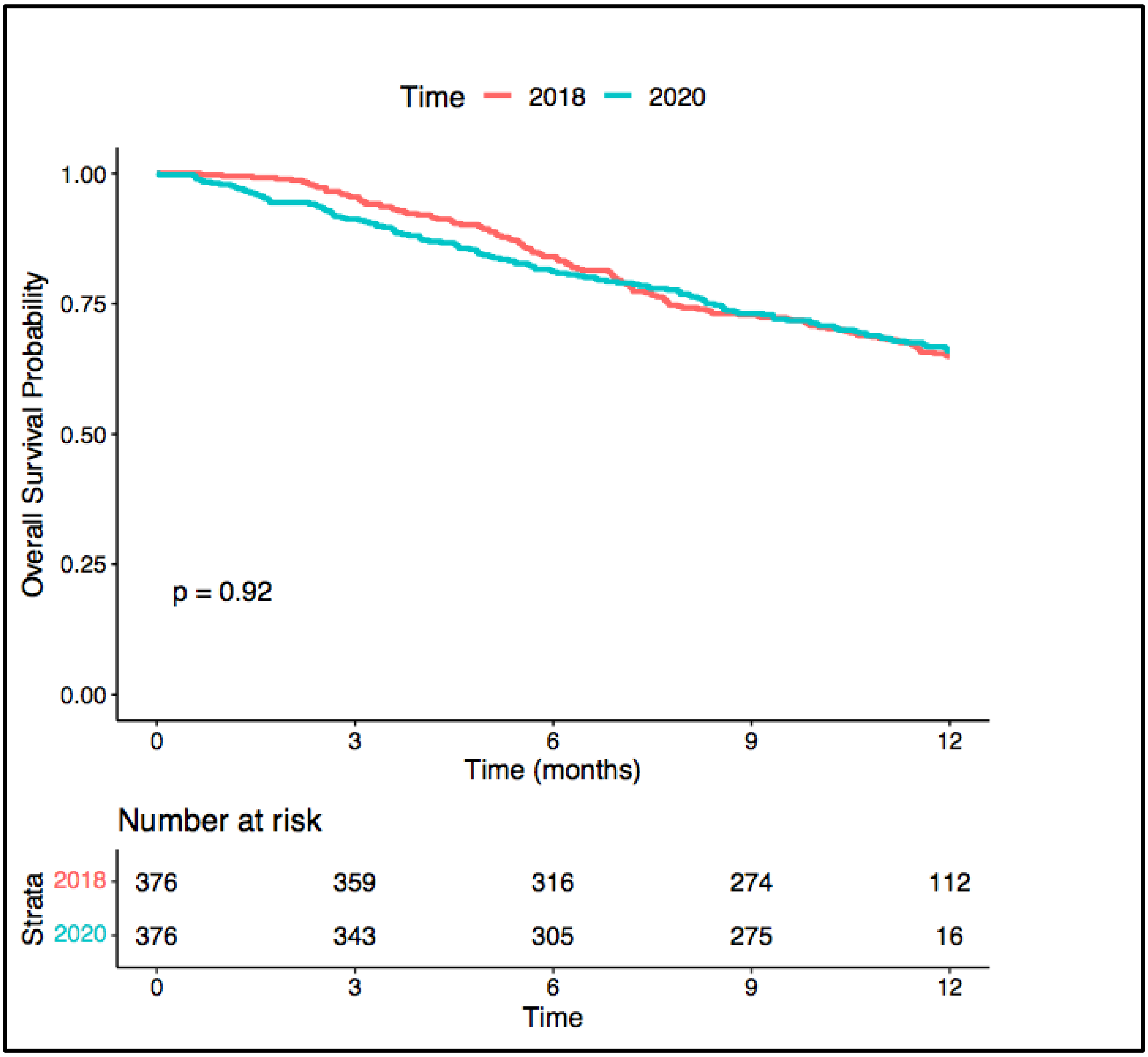

Supplement: Supplementary file 2 — Figure S2. [file CAM4-12-17603-s001.pdf]
