# Supplementary material for: Does COVID‐19 pandemic impact cancer outcomes in metastatic setting? A comparative cohort study among metastatic patients treated at day care hospital
Source: Cancer Med. 2023 Jul 26;12(17):17603–12. doi: 10.1002/cam4.6378 (PMC10523941; doi:10.1002/cam4.6378)
Supplement: Supplementary file 3 — Table S1. [file CAM4-12-17603-s004.pdf]

Supplementary Table 1. Characteristics of the screened population (N = 878)

	2018 cohort<br>N = 501		2020 cohort<br>N = 377		P value
**Gender**					0.11
Women	328	65.5%	226	59.9%	
Men	173	34.5%	151	40.1%	
**Age[a] (years)**					
Median (Q1-Q3)	66.2	(58.4 - 72.3)	65.5	(58.5 - 71.8)	0.5
>70	176	35.1%	126	33.4%	0.65
**Diagnostic delay (months)**					
Median (Q1-Q3)	14	(4.3 - 32.8)	14.9	(5.5-33.5)	0.33
> 24	175	34.9%	128	34.0%	0.82
**Location**					**<0.001**
Breast	161	32.1%	108	28.6%	
Digestive	131	26.1%	93	24.7%	
Gynaecological	75	15.0%	38	10.1%	
Lung	76	15.2%	82	21.8%	
Urogenital	46	9.2%	25	6.6%	
Head and Neck	12	2.4%	31	8.2%	
**Performance status**					0.99
ECOG PS 0-1	430	86.7%	327	87.0%	
ECOG PS 2-3	66	13.3%	49	13.0%	
Missing	5		1		
**BMI[b] (kg/m^2)**					
Median (Q1-Q3)	24.6	(21.0 - 28.1)	24.7	(21.6 - 27.9)	0.79
**Ongoing treatment at inclusion**					0.34
Line 1	238	47.5%	193	51.3%	
Line 2	132	26.3%	100	26.6%	
Line 3	62	12.4%	47	12.5%	
Line ≥ 4	69	13.8%	37	9.8%	
**Ongoing treatment protocol**					**0.003**
Chemotherapy	267	53.3%	181	48.0%	
Chemotherapy + targeted therapy	126	25.1%	76	20.2%	
Immunotherapy	38	7.6%	53	14.1%	
Targeted therapy	70	14.0%	67	17.8%	

[a]: age at inclusion
[b]: body mass index
In bold significant results
