# Supplementary material for: Does COVID‐19 pandemic impact cancer outcomes in metastatic setting? A comparative cohort study among metastatic patients treated at day care hospital
Source: Cancer Med. 2023 Jul 26;12(17):17603–12. doi: 10.1002/cam4.6378 (PMC10523941; doi:10.1002/cam4.6378)
Supplement: Supplementary file 4 — Table S2. [file CAM4-12-17603-s002.pdf]

Supplementary Table 2. Univariable and multivariable analysis for OS in the analysed population

	Univariable analysis			Multivariable analysis		
	HR	95% CI	P value	HR	95% CI	P value
**Gender**<br>*Reference: Female*	1.59	[1.24-2.03]	**<0.001**	1.13	[0.81-1.57]	0.48
**Performance status**<br>*Reference: ECOG PS 0-1*	2.06	[1.51-2.80]	**<0.001**	1.95	[1.40-2.70]	**<0.001**
**BMI[a] (kg/m^2)**			**0.030**			0.071
0-20	1	-		1	-	
20-25	0.89	[0.63-1.24]		0.89	[0.63-1.27]	
25 +	0.67	[0.48-0.94]		0.69	[0.49-0.99]	
**Age (years)**[b]<br>*Reference: < 70*	1.13	[0.87-1.45]	0.36	1.07	[0.82-1.39]	0.63
**Time from diagnosis (months)**<br>*Reference: < 24*	0.64	[0.49-0.84]	**0.001**			
**Number of treatment lines**	1.24	[1.16-1.34]	**<0.001**	1.25	[1.14-1.37]	**<0.001**
**Primary tumour localisation**			**<0.001**			**<0.001**
Breast	1	-		1	-	
Digestive	2.01	[1.42-2.83]		2.60	[1.61-4.21]	
Gynaecological	1.63	[1.03-2.58]		1.79	[1.09-2.93]	
Lung	1.81	[1.26-2.62]		2.71	[1.67-4.38]	
Urogenital	1.68	[0.98-2.85]		1.73	[0.91-3.29]	
Head and Neck	2.59	[1.57-4.28]		3.61	[1.87-6.96]	
**Treatment protocol**			**<0.001**			**<0.001**
Chemotherapy	1	-		1	-	
Chemo + targeted therapy	0.62	[0.45-0.84]		0.62	[0.44-0.88]	
Immunotherapy	0.35	[0.22-0.57]		0.31	[0.18-0.52]	
Targeted therapy	0.17	[0.10-0.30]		0.39	[0.22-0.71]	
**Time period (2018 vs 2020)**<br>*Reference: 2018*	0.99	[0.77-1.26]	0.92	0.91	[0.71-1.16]	0.44

[a]: body mass index
[b] : age at inclusion
In bold significant results
